# An auxiliary diagnosis model for the pathological classification of cervical cancer based on radiomics biomarkers

**DOI:** 10.3389/fgene.2026.1685927

**Published:** 2026-03-11

**Authors:** Mei Wang, Yu Cao, Mengchen Zhu, Peilin Zhao, Qin Zhou, Hongxiang Lan, Jizhao Liu, Junqiang Lei

**Affiliations:** 1 The First Clinical Medical College, Lanzhou University, Lanzhou, China; 2 Department of Obstetrics and Gynecology, The First Hospital of Lanzhou University, Lanzhou, China; 3 The Second Clinical Medical College, Lanzhou University, Lanzhou, China; 4 School of Electronic and Information Engineering, Lanzhou Jiaotong University, Lanzhou, China; 5 School of Information Science and Engineering, Lanzhou University, Lanzhou, China; 6 Gansu Province Clinical Research Center for Radiology Imaging, The First Hospital of Lanzhou University, Lanzhou, China; 7 Intelligent Imaging Medical Engineering Research Center of Gansu Province, The First Hospital of Lanzhou University, Lanzhou, China

**Keywords:** biomarkers, cervical cancer, medical image segmentation, pathological classification, radiomics

## Abstract

**Introduction:**

Cervical cancer remains a major global health burden, and accurate pathological classification is essential for personalized treatment planning. However, conventional radiomics studies often rely on manual lesion delineation and are limited in extracting meaningful imaging biomarkers from heterogeneous cervical cancer lesions.

**Methods:**

We proposed a convolutional recurrent feature extraction (CRFE)-based automatic segmentation framework for cervical cancer MRI images and developed histogram-based imaging features reflecting lesion pixel concentration trends. These features were integrated with conventional radiomics and clinical features. Feature engineering and machine learning classifiers, including random forest (RF), XGBoost, support vector machine, and logistic regression, were evaluated to construct an auxiliary diagnostic model for pathological classification. The dataset included 114 patients with cervical cancer who underwent MRI examinations.

**Results:**

The CRFE segmentation model achieved an Intersection over Union (IoU) of 0.9443, a Dice coefficient of 0.5980, and an F1-score of 0.7085. Feature selection retained 30 key imaging biomarkers, including the median of the histogram, GLSZM large-area low gray-level emphasis (LoG, *σ* = 2.0*mm*, 3D), and GLRLM long-run low gray-level emphasis (LoG, *σ* = 2.0*mm*, 3D). Among the evaluated classifiers, the RF model achieved the best performance, with an accuracy of 87.27% and an F1-score of 86.91% in pathological classification.

**Discussion:**

The proposed deep learning–radiomics framework enables accurate lesion segmentation and effective pathological classification of cervical cancer. This auxiliary diagnostic model may reduce unnecessary invasive procedures and improve early screening and clinical decision-making.

## 1 Introduction

Radiomics, an emerging medical image analysis technology, objectively describes the shape, texture, density, and spatial distribution of tumors (Mayerhoefer et al., 2020; Lambin et al., 2017) based on high-throughput quantitative features extracted from computed tomography (CT) (Berenguer et al., 2018), magnetic resonance imaging (MRI), or positron emission computed tomography (PET) (Cook et al., 2014) images and provides a detailed description of the phenotypic information of tumors (Gillies et al., 2016). For cervical cancer, MRI radiomics can effectively distinguish between cervical squamous cell carcinoma and adenocarcinoma and predict lymph-node metastasis (Follen et al., 2003). However, in existing radiomics research of cervical cancer, the delineation of the lesion area heavily relies on manual labeling (Oussahi et al., 2024), which cannot effectively analyze large-scale image data (Zabihollahy et al., 2022). Furthermore, the regional heterogeneity of cervical cancer lesions is high, and the image features are difficult to reliably extract (Kumar et al., 2012). This hinders the construction of effective biomarkers and makes rapid diagnosis and early screening difficult (Shah et al., 2022; Kessler, 2017). In order to solve this problem, we first designed a deep learning-based lesion area extraction model for cervical cancer. Specifically, based on the U-Net architecture (Zhou et al., 2018), we combined convolutional networks, a recurrent structure, and an attention mechanism to form the convolutional recurrent feature extraction (CRFE) model, which improves contextual modeling and key-region recognition, thereby enabling automatic segmentation of cervical cancer lesions. On this basis, we designed a variety of imaging features for the lesion area, including gray-level run length matrix–long run low gray-level emphasis (GLRLM–LRLGLE), gray-level size zone matrix–large area high gray-level (GLSZM–LAHGL), and median of histogram, and added these features to existing radiomics features. Combined with clinical features, feature screening was performed to identify biomarkers for the diagnosis of cervical cancer. Finally, an auxiliary diagnosis model of cervical cancer pathological classification based on the biomarkers of the imaging group was formed. The main contributions of this study are summarized as follows:

1. We designed a deep learning lesion region extraction method for cervical cancer images. Based on the U-Net architecture (Zhou et al., 2018), our method integrates convolutional networks, cyclic structures, and attention mechanisms (Niu et al., 2021; Brauwers and Frasincar, 2021) and uses Dice loss and cross-entropy loss to construct the CRFE model. The Intersection over Union (IoU) of the model is 0.9443, the Dice coefficient is 0.5980, and the F1-score is 0.7085, which can accurately extract the cervical cancer lesion area.
2. Based on characteristics of cervical cancer lesion area images, we designed a series of imaging features, including the median of histogram, to perform more comprehensive radiomics feature extraction.
3. We added designed features to the existing radiomics features and integrated the clinical features to construct an auxiliary diagnosis model of cervical cancer pathological classification. The results show that the median of the histogram, GLSZM large area low gray-level emphasis (LoG, σ=2.0mm , 3D), GLRLM long run low gray-level emphasis (LoG, σ=2.0mm , 3D), gray-level dependence matrix (GLDM) dependence variance (LoG, σ=1.0mm , 3D), and 27 other features have a better ability to predict cervical cancer lesions than clinical features alone. This may help reduce unnecessary clinical examinations and improve the efficiency of early screening and clinical diagnosis of cervical cancer.

## 2 Materials and methods

### 2.1 Dataset

This study is a single-center retrospective study (Talari and Goyal, 2020) and was approved by the Ethics Committee of the First Hospital of Lanzhou University (with informed consent waived due to anonymous data analysis). A total of 114 patients with cervical cancer who were treated in our hospital from January 2022 to December 2024 were included in the study. All subjects underwent MRI examination before the initial treatment.

The inclusion criteria included the following:

1. Patients with cervical cancer diagnosed by pathology as squamous cell carcinoma or adenocarcinoma.
2. FIGO (International Federation of Gynecology and Obstetrics) staging (Bhatla et al., 2019) was IA to IIIC.
3. Newly diagnosed patients without surgical treatment.
4. The age ranged from 28 to 80 years old, with a Karnofsky (Péus et al., 2013) score of ≥70 points.
5. Patients who underwent standard MRI acquisitions, including T2-weighted imaging (T2WI), diffusion-weighted imaging (DWI), and apparent diffusion coefficient (ADC).

FIGO staging was used to classify the tumor extent. Stage IA represents microscopic lesions that are confined to the cervix, whereas stage IIIC indicates pelvic and/or para-aortic lymph-node metastasis. Stage IIIC disease implies more widespread cancer involvement. Because nodal metastasis can alter the tissue contrast on MRI, stage IIIC lesions may be more distinguishable in the proposed model. The Karnofsky Performance Status (KPS) score assesses a patient’s functional ability, ranging from 0 to 100; with scores ≥ 70 indicating that the patient can care for themselves.

The exclusion criteria included the following:

1. Patients who have undergone surgery / other systemic treatment.
2. Patients with poor MRI image quality / missing key sequences.
3. Patients with severe infectious diseases / autoimmune diseases / blood diseases / liver diseases / other malignant tumors.
4. Patients with extensive metastatic disease beyond the pelvic region.

The median age of the included patients was 51 years (range: 28–80 years). All images were acquired using the same MRI system (Siemens 3.0-T MAGNETOM Skyra and Philips Ingenia 3.0-T). Image quality control consisted of standardized slice thickness, field-of-view consistency, signal-to-noise ratio assessment, and the exclusion of studies with motion artifacts (Table 1).

**TABLE 1:** Summary of the baseline characteristics, clinicopathological features, and treatment methods of the patients (n = 114). The table shows the age, HPV infection status, lymph-node metastasis, lymphatic vascular invasion, cervical infiltration depth, clinical stage, and chemotherapy and radiotherapy of 114 patients included in the study. The data are presented as the mean ± standard deviation, median (range), frequency (percentage), and 95% confidence interval. Patients with unknown FIGO stage were included to avoid unnecessary exclusion and potential selection bias. Treatment information was recorded as baseline clinical data and was not used as a predictive variable in model training.

Characteristic	Category/Statistic	Value (n = 114)	Percentage	95% Confidence interval
Age	Mean ± SD	51.2 ± 8.7	​	(49.6, 52.8)
	Median (range)	51 (28–80)	​	​
HPV status	Positive	78	68.40%	(59.2%, 76.8%)
	Negative	9	7.90%	(3.7%, 14.5%)
	Not reported	27	23.70%	(16.2%, 32.7%)
Lymph node metastasis	Absent	95	83.30%	(75.3%, 89.6%)
	Present	19	16.70%	(10.4%, 24.7%)
Lymphovascular invasion	Absent	102	89.50%	(82.3%, 94.4%)
	Present	12	10.50%	(5.6%, 17.7%)
Depth of cervical invasion (cm)	Mean ± SD	0.54 ± 0.28	​	(0.49, 0.59)
	Median (range)	0.5 (0.1–1.0)	​	​
FIGO stage	Stage I (IA/IB)	48	42.10%	(32.9%, 51.8%)
	Stage II (IIA/IIB)	51	44.70%	(35.5%, 54.3%)
	Stage III/IV	12	10.50%	(5.6%, 17.7%)
	Unknown	3	2.60%	(0.6%, 7.5%)
Treatment	Chemotherapy (yes)	88	77.20%	(68.5%, 84.6%)
	Radiotherapy (yes)	58	50.90%	(41.4%, 60.3%)

### 2.2 CRFE segmentation model

In order to realize the automatic and accurate segmentation of the lesion area in cervical cancer images, this study proposes a segmentation model based on the CRFE architecture. The model utilizes the traditional convolutional neural network (CNN) (Zhao et al., 2024) to extract spatial features, combines the loop structure to capture the contextual connections (Chen et al., 2016; Zuo et al., 2016) in the image, and strengthens the key region features through the attention mechanism. Strengthening key regional features enhances the representation of clinically relevant areas, whereas biasing the model toward specific key features may reduce generalizability. The attention mechanism was introduced to adaptively weight important lesion regions without overemphasizing noise, thereby improving both interpretability and segmentation accuracy. The implementation process of the model includes three aspects: data preprocessing, the CRFE framework, and the training strategy.

#### 2.2.1 Data preprocessing

In order to ensure a unified input data format and enhance the generalization ability of the model, we preprocessed the original MRI image data of 141 patients with cervical cancer from the Department of Gynecology, First Clinical Medical College of Lanzhou University (114 patients had complete clinical and imaging data). MRI images were obtained using two different 3.0-T magnetic resonance scanners (Siemens MAGNETOM Skyra and Philips Ingenia). Analysis utilized three sequences, including T2-weighted imaging (T2WI), diffusion-weighted imaging (DWI), and their corresponding apparent diffusion coefficient (ADC) maps. First, all images are resampled to a uniform resolution to ensure the consistency of the input images on the spatial scale (Anthony Parker et al., 2007; Yu, 2002). Subsequently, the intensity standardization is carried out, and the z-score standardization (Curtis et al., 2016; Gao et al., 2019) is applied to the image to eliminate the difference in grayscale distribution caused by different acquisition equipment and scanning parameters (Saravanan, 2010; Lissner et al., 2012). In order to expand sample diversity and improve robustness (Kitano, 2004; Gowal et al., 2021), a random data enhancement strategy was adopted. It includes random rotation of the image in the range of ± 15°, random flipping of the image in the horizontal and vertical directions (Zhong et al., 2020; Takahashi et al., 2019), random scaling of the image in the range of 0.9–1.1 times, adding Gaussian noise (Gedraite and Hadad, 2011; Ahmad B. et al., 2022; Pyatykh et al., 2012) with a standard deviation of 0.01 to improve the robustness of the model to the noise, and dividing the dataset by 70% (99 cases) of the training set, 15% (21 cases) of the validation set, and 15% (21 cases) of the test set.

#### 2.2.2 CRFE framework

The CRFE segmentation framework was constructed by adopting the encoder–recursive feature extraction module–decoder structure. The encoder part is responsible for extracting the multi-scale spatial features of the input MRI image, and the input layer accepts a two-dimensional image with a size of 256 × 256. The three convolution blocks adopt 32 3 × 3 convolution kernels, 64 3 × 3 convolution kernels, and 128 3 × 3 convolution kernels, respectively, and cooperate with the ReLU activation function ReLU(x)=max(0,x) to extract image features from low to high (Schmidt-Hieber, 2020). By using a 2 × 2 pooling window, downsampling is carried out (Wu et al., 2023), the feature map size is reduced, and the feature invariance is enhanced. To incorporate global context information, the module also enhances feature extraction by introducing a cyclic structure. Based on the features extracted by convolutional coding, 256 long short-term memory (LSTM) units are set up to learn the local and global spatio-temporal dependencies in the image. In addition, the module captures both forward and reverse information to fully model the complex context dependencies that may exist in the image. Notably, we introduce the attention module after the loop layer and assign adaptive weights according to the importance of different regions to highlight the features of key regions and alleviate the problem of feature redundancy (Ma et al., 2019). The spatial attention module can establish a rich context model on local features and encode wider context information into local features, thereby enhancing its representation ability.

Given the local feature $A\in R^{C\times H\times W}$ (where A denotes the input local convolutional feature map and C , H , and W represent the number of channels and the spatial height and width, respectively), it is first passed through a convolutional layer to generate two new feature maps B and C , each of size $R^{C\times H\times W}$ . These feature maps are then reshaped to $R^{C\times N}$ , where N=H×W denotes the total number of pixels. This reshaping converts the spatial dimensions into a single dimension, enabling pixel-wise computation in the subsequent attention operation. After reshaping, matrix multiplication is performed between B and C , and the result is normalized using a softmax layer to produce the spatial attention map (Hu et al., 2018).

$$S\in R^{C\times N},$$ (1)

$$S_{ij}=\frac{\exp\left(B_{i}\cdot C_{j}\right)}{\sum_{i-1}^{N}\exp\left(B_{i}\cdot C_{j}\right)}.$$ (2)

In Equation 2, $S_{ij}$ is the influence of position i on position j. A more similar feature representation of the two locations contributes to a greater correlation between them. In parallel, the original feature map A is passed through an additional convolutional layer to generate another feature map $D\in R^{C\times H\times W}$ , which is likewise reshaped into $R^{C\times N}$ to match the dimensionality required for the attention computation. Subsequently, matrix multiplication is performed between $D^{T}$ and the spatial attention map S obtained from Equations 1, 2, and the resulting feature representation is reshaped back to $R^{C\times H\times W}$ . Finally, as described in Equation 3, the output feature is obtained by multiplying the aggregated context feature by the learnable coefficient α and adding it element-wise to the original feature A , producing the following attention-enhanced feature:

$$E_{j}=\alpha \overset{N}{\underset{i=1}{\sum }}\left(S_{ji}\cdot D_{i}\right)+A_{j}.$$ (3)

Here, $E_{j}$ denotes the output feature vector at position j , which is enhanced by global contextual information; $S_{ji}$ denotes the attention weight indicating the contribution of position i to j ; $D_{i}$ denotes the context feature at position i ; and $A_{j}$ denotes the original local feature at position j , which is used to establish a residual connection. Here, α is initialized to 0, and more weights are gradually assigned, and it can be inferred that the result feature E at each position is the weighted sum of the features at all the positions and the original features. Therefore, by incorporating the global context view and selectively aggregating the features according to the spatial attention map, similar semantic features are enhanced, thereby improving intra-class compactness and semantic consistency. In the decoder part, 2×2 up-sampling is used to enlarge the low-resolution feature map generated by the encoder. The up-sampled feature map is refined using 64 3×3 convolution kernels, and 32 3×3 convolution kernels are used to further fuse local information. The 1×1 convolution kernel is used to generate a single-channel segmentation probability map, and the final segmentation result is output by the sigmoid activation function, as shown in Equation 4 (Narayan, 1997). The original image size is gradually restored by up-sampling and convolution operations, and the final segmentation result is generated.

$$\sigma \left(x\right)=\frac{1}{1+e^{-x}},$$ (4)

where σ(x) represents the sigmoid activation function, x denotes the input value to the activation unit, and e is Euler’s number, which is the base of the natural logarithm. The sigmoid function maps any real-valued input into the range (0, 1).

**FIGURE 1:**
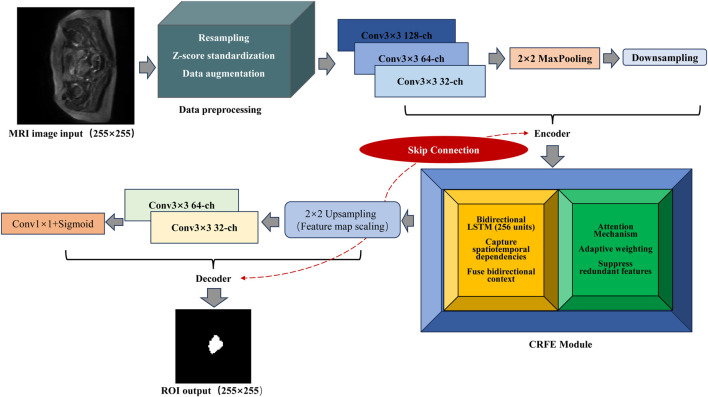
Convolutional recurrent feature extraction framework. The input is a 255×255 MRI image, and after data preprocessing (resampling, Z-score standardization, and data enhancement), it enters the encoder (including convolutional layer Conv 3×3 128/64/32-ch, 2×2 maximum pooling down-sampling, and skip connection). Skip connections refer to feature map connections between corresponding encoder and decoder layers to preserve spatial information. The feature then captures spatio-temporal dependencies and fuses bi-directional context information through a module containing bi-directional LSTM (256 units) and then adaptively weights key lesion-related features through the attention mechanism to suppress redundant features. LSTM denotes a long short-term memory network used to capture contextual dependencies in the feature maps. The decoder part includes upsampling (2×2) , convolution layer (Conv 3×3 64/32-ch, and Conv 1×1 + sigmoid), and finally the output ROI segmentation map of the same size (255×255) . The independent CRFE module is also marked in the figure.

The framework fully integrates the information from convolution features and recurrent modules based on MRI images and highlights key features using an attention mechanism, effectively improving the segmentation accuracy and robustness of cervical cancer lesions. The model framework is shown in Figure 1.

#### 2.2.3 Model training strategy

In order to ensure that the model converges quickly and stably in the segmentation task, a series of optimized training strategies were adopted in this study. First, in the design of loss function, Dice loss (Zhao et al., 2020) and cross-entropy loss (Zhang and Sabuncu, 2018) are used to balance the accuracy of region overlap and pixel classification. Specifically, the Dice loss is defined as shown in Equation 5:

$$Diceloss=1-\frac{2|X\cap Y|+\varepsilon }{|X|+|Y|+\varepsilon },$$ (5)

where |X∩Y| denotes the number of pixels correctly predicted as the lesion region, |X| and |Y| represent the total number of predicted and ground-truth positive pixels, respectively, and ε is a small constant added for numerical stability.

The cross-entropy loss is defined as shown in Equation 6:

$$CrossEntropyLoss=-\sum y\cdot \log\left(p\right),$$ (6)

where y is the ground-truth binary label, p is the predicted probability for the corresponding class, and log(⋅) denotes the natural logarithm. This loss penalizes incorrect high-confidence predictions.

The final combined loss function is defined in Equation 7:

$$L_{\text{total}}=\overset{N_{\text{module}}}{\underset{i=1}{\sum }}L_{\text{module }i}^{\left(\text{binary}\right)}+\lambda \cdot \overset{N_{\text{stand}}^{\prime }}{\underset{j=1}{\sum }}L_{\text{global }j}^{\left(\text{binary}\right)}+L_{\text{size}}^{\left(\text{binary}\right)}.$$ (7)

Here, $N_{\text{module}}$ represents the number of sub-modules (such as encoder/decoder), and $L_{\text{module }i}^{\left(\text{binary}\right)}$ is the binary segmentation loss (Dice/cross entropy) of each module, which supervises local feature learning. $N_{\text{stand}}^{\prime }$ represents the number of global constraint terms, and $L_{\text{module }j}^{\left(\text{global}\right)}$ is weighted by the hyperparameter λ to ensure the spatial consistency of prediction. $L_{\text{size}}^{\left(\text{binary}\right)}$ is based on the prior clinical size of cervical cancer lesions (average diameter 1 cm–4 cm) (Horn et al., 2007), and any prediction that deviates from the medically reasonable range is penalized. This design forces the model to distinguish the lesion (positive class) from the surrounding tissue (background).

For the selection of the optimizer, this study used the Adam optimizer (Zhang, 2018), with an initial learning rate of 0.001. In order to ensure smooth parameter updates during training, the cosine-annealing strategy (Zhang et al., 2024) was used to gradually decay the learning rate. The cosine-annealing learning rate schedule is defined in Equation 8:

$$\eta_{t}=\eta_{\min}^{i}+\frac{1}{2}\left(\eta_{\max}^{i}-\eta_{\min}^{i}\right)\left(1+\cos\left(\frac{T_{\mathrm{cur}}}{T_{i}}\pi \right)\right).$$ (8)

Here, $\eta_{t}$ denotes the learning rate used at iteration t . The symbols $\eta_{\min}^{i}$ and $\eta_{\max}^{i}$ represent the minimum and maximum learning rates within the i th cosine annealing cycle, respectively. The variable $T_{\mathrm{cur}}$ refers to the current number of iterations completed in the ongoing cycle, while $T_{i}$ denotes the total number of iterations assigned to the i th cycle. The cosine function cos(x) is utilized to generate a smooth learning rate decay curve, and π denotes the mathematical constant pi. Together, these components construct a cosine-annealing schedule that gradually reduces the learning rate from $\eta_{\max}^{i}$ to $\eta_{\min}^{i}$ within each cycle, thereby facilitating stable convergence and improving the robustness of the training process.

During the training process, the batch size is set to 4, which not only ensures the rational use of video memory but also ensures the stability of training. The number of training rounds (epochs) is set to 100 to ensure that the model can fully converge. In addition, in order to prevent over-fitting, an early stopping strategy based on the Dice coefficient of the validation set was adopted. Specifically, the monitoring index is the Dice coefficient on the validation set. When the improvement of the Dice coefficient in 10 consecutive epochs is less than 0.001, the training will stop in advance, thus avoiding the occurrence of overfitting (Table 2).

**TABLE 2:** Training strategy. lr, learning rate.

Component	Configuration details
Loss function	$L_{total}=\sum_{i=1}^{N_{module}}L_{modulei}^{\left(binary\right)}+\lambda \cdot \sum_{j=1}^{N_{stand}}L_{globalj}^{\left(binary\right)}+L_{size}^{\left(binary\right)}$
Optimizer	Adam (lr = 0.001)
Learning rate adjustments	Cosine-annealing strategy
Batch size	4
Epochs	100
Early stop strategy	There was no improvement in the Dice coefficient of the validation set in 10 rounds

### 2.3 Biomarker construction and radiomics analysis

#### 2.3.1 Data preparation and processing

In this study, a multi-source fusion dataset was constructed, which integrated three types of complementary feature information: first, clinical data obtained from the clinical process, including patients’ clinical information and pathological test indicators; second, histogram features extracted from medical images that reflect the gray distribution of the image; third, high-dimensional radiomics features extracted by radiomics analysis technology, covering multi-level image features such as texture, shape, and transformation. All data are merged with the patient’s unique identification as the matching key to ensure the consistency of the model data.

In the data preprocessing stage, all numerical features are first aggregated and normalized to eliminate missing and abnormal values, and downsampling (Zhou, 2020) is used to fill missing values and improve data quality. Based on the three original binary pathological annotation variables, a new multi-classification label ‘Pathologic_typing’ is constructed to characterize the specific pathological classification status of patients and provide a unified prediction target for training downstream multi-classification models.

#### 2.3.2 Feature extraction and screening

In feature extraction and screening, the features of cervical cancer lesions in MRI images of 55 patients with detailed clinical information were extracted by OpenCV (cv2) and the PyRadiomics library. In order to improve the generalization ability of the model and reduce dimension redundancy, a systematic feature selection strategy was used to screen and optimize the input features. First, by evaluating combinations of different numbers of features (ranging from 5 to 100), the performance of each combination was assessed under a cross-validation framework (Zhong et al., 2010) to automatically determine the optimal number of features. Three complementary feature selection methods are further introduced:

1. Based on the model-based selection method, the feature importance score generated by random forest (Qi, 2012) is used to screen high-weight features.
2. Based on univariate selection, analysis of variance (ANOVA) F-test was used to measure the linear dependence between each feature and the target variable (Chen et al., 2018).
3. Recursive feature elimination (RFE) (Darst et al., 2018), which gradually converges to the optimal feature subset, was used to iteratively train and remove the least important features.

All features are standardized using the StandardScaler method (Ahmad G. et al., 2022) before model training so that they conform to the standard normal distribution hypothesis with a mean of 0 and a variance of 1, thereby improving the model’s convergence speed and performance stability.

#### 2.3.3 Model training and optimization

This study compared and evaluated the performance of four mainstream classification models in the pathological classification task of cervical cancer, including random forest (RF), extreme gradient boosting (XGBoost, XGB) (Chen et al., 2015), support vector machine (SVM) (Shan, 2016), and logistic regression (LR) (Christodoulou et al., 2019). In order to address the problem of uneven sample distribution, the synthetic minority over-sampling technique (SMOTE) (Chawla et al., 2002) was used to oversample the minority classes in the training set, generating synthetic samples to enhance the model’s ability to recognize underrepresented categories. During the training process, the grid search (GridSearchCV) is used to tune the key hyperparameters of each model (Ahmad G. et al., 2022). Each model presets a specific parameter search space and performs performance evaluation through 5-fold cross-validation to ensure the optimality of the hyperparameter combination in generalization ability (Browne, 2000).

#### 2.3.4 Model evaluation and validation

The performance of the final model is verified in the test set using the following metrics, as defined in Equations 9–12:

$$Accuracy=\frac{TP+TN}{TP+TN+FP+FN},$$ (9)

$$Precision=\frac{TP}{TP+FP},$$ (10)

$$testRecall=\frac{TP}{TP+FN}.$$ (11)

True positive (TP) refers to the number of positive samples that are correctly identified as positive, whereas true negative (TN) denotes the number of negative samples correctly predicted as negative. False positive (FP) represents the number of negative samples that are incorrectly classified as positive, and false negative (FN) refers to the number of positive samples that are mistakenly classified as negative. Based on these quantities, the F1-score is adopted as a comprehensive performance indicator.

$$F_{1}=2\times \frac{\text{Precision}\times \text{Recall}}{\text{Precision}+\text{Recall}}=\frac{2TP}{2TP+FP+FN}.$$ (12)

The F1-score is defined as the harmonic mean of precision and recall, providing a balanced evaluation that simultaneously accounts for both FPs and FNs. Due to its robustness in handling class imbalance, the F1-score offers a more reliable measure of model performance than accuracy alone.

The evaluation metrics, including accuracy, precision, recall, and the F1-score, were calculated (Naidu et al., 2023), and the prediction performance of the model for each category was further analyzed using the confusion matrix. In addition, feature importance analysis was conducted to identify and visualize the features that contributed most to model’s predictions. On this basis, the importance distribution of clinical features and radiomics features was statistically analyzed to reveal their complementary role in the pathological classification task.

### 2.4 Implementation details

The research was conducted on the Ubuntu 20.04 LTS operating system, using a software environment comprising Python (3.8.10), CUDA (11.7), cuDNN (8.5.0), and the deep learning framework PyTorch (1.13.1) to train and evaluate the model.

## 3 Result

In this section, we present the experimental results of the proposed framework, which aims to (1) automatically segment cervical cancer lesions using the CRFE model and (2) construct an auxiliary diagnostic model for pathological classification based on radiomics biomarkers. The results are organized into two parts: the performance evaluation of the CRFE segmentation model and the effectiveness of the auxiliary pathological diagnosis model.

### 3.1 CRFE segmentation model

**FIGURE 2:**
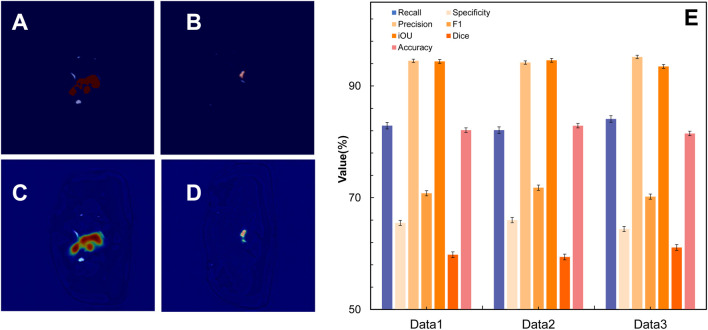
Segmentation performance of the CRFE model on cervical cancer MRI images. **(A)** Standard lesion contour of a certain MRI slice; **(C)** lesion contour obtained by the model after segmentation of the slice; **(B)** standard lesion area of all the other slices of the patient; **(D)** lesion contour obtained by the model after segmentation of the slice; **(E)** performance index of the CRFE model on three test sets of MRI images.

In terms of segmentation performance indicators, the IoU value of the model is 0.9443, the Dice coefficient is 0.5980, and the F1-score is 0.7085. Among these metrics, the IoU value of 0.9443 is close to the ideal value of 1.0, indicating excellent overall spatial overlap between the predicted and reference lesion regions. Although the Dice coefficient and F1-score are lower, they remain within an acceptable range for medical image segmentation tasks involving complex lesion boundaries. In terms of classification performance, the accuracy rate is 0.8000, the precision rate is 0.9450, the recall rate is 0.8290, and the specificity is 0.6550. In terms of performance indicators, the mean absolute error (MAE) is 70.85, the Hausdorff distance (HD) is 16.46, and the inference time is 450.00 ms. In addition, the model evaluation program generates artificial segmentations of random samples and image comparisons of lesion areas based on the CRFE model’s segmentation. Figures 2A–D present the qualitative segmentation comparisons, while Figure 2E summarizes the quantitative performance metrics.

### 3.2 Auxiliary diagnosis model of pathological classification of cervical cancer

#### 3.2.1 Features extracted

**FIGURE 3:**
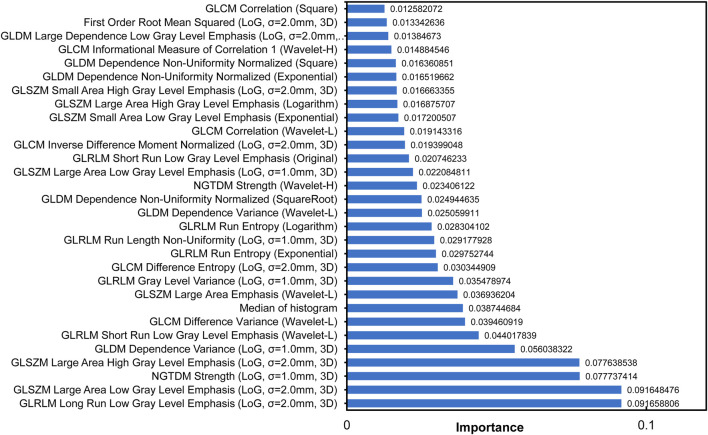
Importance ranking of radiomics features. The plot displays the normalized importance scores of various radiomics features extracted from medical images, including texture features (GLCM, GLDM, GLSZM, GLRLM, and NGTDM) and first-order statistics. Features were processed using different filters with specified parameters ( σ=1.0−−2.0mm , 3D). Details of the applied filters and feature definitions are provided in the Methods section. The lower-ranked features (e.g., “GLCM correlation (square)”) show higher importance values (ranging up to 0.0917), indicating their potential diagnostic relevance.

**FIGURE 4:**
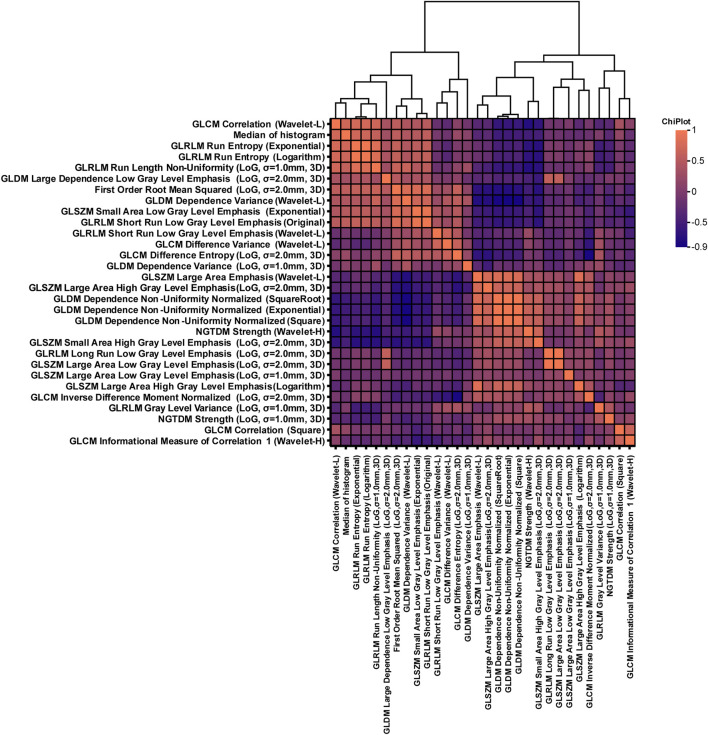
Correlation heatmap and hierarchical clustering analysis of texture features of radiomics. The horizontal and vertical axes in the image are the extracted radiomics texture features, including GLCM, GLRLM, GLSZM, GLDZM, and NGTDM. The figure shows the Pearson correlation coefficient between the representative features of the color of the heatmap (red shows the positive correlation, blue shows the negative correlation, and the color range is 0.9–0.5); the tree diagram represents a hierarchical clustering result based on feature correlation, which reflects the similarity clustering relationship between features, and is displayed above the heatmap.

Through the integration of multi-source data and the extraction of cervical cancer lesion features, a total of 868 features were obtained. These included clinical features, histogram-based features, morphological features (e.g., Elongation and MajorAxisLength), GLCM (gray-level co-occurrence matrix) features; GLSZM (gray-level size zone matrix) features, GLRLM (gray-level run length matrix) features; GLDM (gray-level dependence matrix) features, NGTDM (neighborhood gray-tone difference matrix) features; and first-order statistical features (Table 3). After feature selection, 30 features with the highest predictive power were retained from the original feature set (Tables 4–6). Analysis of feature importance and feature correlation revealed that these 30 selected features included a histogram-based feature, namely, the median, which represents the median pixel intensity and ranked eighth in importance among all the features. The feature correlation heatmap more clearly illustrates the relationships among the features, indicating that the 30 selected features—including histogram features derived from MRI images of the lesion regions—are closely associated with the auxiliary diagnosis of cervical cancer (Figures 3, 4). The cutoff at the top 30 features was determined based on the distribution of normalized feature importance scores. As shown in Figure 3, the importance values exhibited a clear inflection point after the 30th feature, with scores decreasing sharply below 0.01. Including additional features beyond this point did not lead to further improvement in classification performance during cross-validation, while increasing the risk of feature redundancy and overfitting. The feature correlation heatmap in Figure 4 shows distinct groups of radiomics features with strong internal correlations. Features derived from the same texture matrices and similar filtering strategies tend to cluster together, indicating shared information content. In particular, GLSZM- and GLRLM-based features form tightly correlated clusters, indicating that they capture complementary aspects of lesion heterogeneity, while first-order and histogram features show weaker correlations with texture-based features, highlighting their independent contribution to the classification model.

**TABLE 3:** Summary of the extracted features used for the auxiliary diagnosis model.

Feature category	Description	Number of features
Clinical features	Patient demographics and pathological indicators, including age, HPV status, FIGO stage, lymph-node metastasis, lymphovascular invasion, and treatment information	8
Morphological features	Shape- and geometry-related features extracted from lesion regions, including volume, surface area, elongation, major axis length, and sphericity	14
First-order statistical features	Intensity-based features describing voxel intensity distributions, such as the mean, median, variance, skewness, kurtosis, and root mean squared values	162
Texture features (GLCM)	Gray-level co-occurrence matrix features characterizing spatial gray-level dependencies, including contrast, correlation, entropy, and homogeneity	216
Texture features (GLRLM)	Gray-level run length matrix features describing the run-length distributions of gray levels	144
Texture features (GLSZM)	Gray-level size zone matrix features quantifying homogeneous gray-level zones	144
Texture features (GLDM)	Gray-level dependence matrix features measuring gray-level dependence patterns	126
Texture features (NGTDM)	Neighborhood gray-tone difference matrix features reflecting local gray-level differences	45
Histogram features	Histogram-based features describing gray-level distribution trends	9
Total	​	**868**

Bold values indicate the total number of extracted features.

**TABLE 4:** Selected important features: part 1.

Feature name	Description
GLRLM long run low gray-level emphasis (LoG, σ = 2.0 mm, 3D)	Emphasis on long continuous segments of low gray-levels, extracted from 3D images after Laplacian of Gaussian filtering with σ = 2.0 mm
GLSZM large area low gray-level emphasis (LoG, σ = 2.0mm, 3D)	Emphasis on large-sized regions with low gray-levels, extracted from 3D images after LoG filtering (σ = 2.0 mm)
NGTDM strength (LoG, σ = 1.0 mm, 3D)	Average gray-tone difference in local neighborhoods, reflecting the intensity of local gray-level variations (σ = 1.0 mm)
GLSZM large area high gray-level emphasis (LoG, σ = 2.0 mm, 3D)	Emphasis on large-sized regions with high gray-levels, extracted after LoG filtering (σ = 2.0 mm)
GLDM dependence variance (LoG, σ = 1.0 mm, 3D)	Variance of gray-level dependencies (correlations at fixed distance) with σ = 1.0 mm
GLRLM short-run low gray-level emphasis (Wavelet-L)	Emphasis on short continuous runs of low gray-levels from low-frequency wavelet components
GLCM difference variance (Wavelet-L)	Variance of gray-level differences, reflecting dispersion of gray-level changes in low-frequency wavelets
Median of histogram	Median value of gray-level histogram, representing central tendency of distribution
GLSZM large area emphasis (Wavelet-L)	Emphasis on large-sized regions (regardless of gray level) from low-frequency wavelet component
GLRLM gray-level variance (LoG, σ = 1.0 mm, 3D)	Variance of gray-levels within individual runs after LoG filtering (σ = 1.0 mm)
GLCM difference entropy (LoG, σ = 2.0 mm, 3D)	Entropy of gray-level differences, measuring uncertainty of variations (σ = 2.0 mm)
GLRLM run entropy (exponential)	Entropy of run length distribution normalized by exponential transformation
GLRLM run length non-uniformity (LoG, σ = 1.0 mm, 3D)	Non-uniformity of run length distribution (dispersion) with σ = 1.0 mm

**TABLE 5:** Selected important features: part 2.

Feature name	Description
GLRLM run entropy (logarithm)	Entropy of run length distribution normalized by logarithmic transformation
GLDM dependence variance (Wavelet-L)	Variance of gray-level dependencies from low-frequency wavelet component
GLDM dependence non-uniformity normalized (square root)	Normalized non-uniformity of dependencies via square-root transformation
NGTDM strength (Wavelet-H)	Average gray-tone difference focusing on fine-grained variations in high-frequency wavelets
GLSZM large-area low gray-level emphasis (LoG, σ = 1.0 mm, 3D)	Emphasis on large low-gray regions at finer scale (σ = 1.0 mm)
GLRLM short-run low gray-level emphasis (original)	Emphasis on short continuous runs of low gray-levels from the original image
GLCM inverse difference moment normalized (LoG, σ = 2.0 mm, 3D)	Normalized inverse difference moment measuring local uniformity (σ = 2.0 mm)
GLCM correlation (Wavelet-L)	Linear correlation coefficient reflecting texture regularity in low-frequency wavelets
GLSZM small area low gray-level emphasis (exponential)	Emphasis on small-sized low-gray regions after exponential transformation
GLSZM large area high gray-level emphasis (logarithm)	Emphasis on large high-gray regions after logarithmic transformation
GLSZM small area high gray-level emphasis (LoG, σ = 2.0 mm, 3D)	Emphasis on small-sized regions with high gray-levels (σ = 2.0 mm)
GLDM dependence non-uniformity normalized (exponential)	Normalized non-uniformity enhanced by exponential transformation
GLDM dependence non-uniformity normalized (square)	Normalized non-uniformity amplified by square transformation

**TABLE 6:** Selected important features: part 3.

Feature name	Description
GLCM informational measure of correlation 1 (Wavelet-H)	Informational measure of linear correlation focusing on fine textures in high-frequency wavelets
GLDM large dependence low gray-level emphasis (LoG, σ = 2.0 mm, 3D)	Emphasis on low-gray dependencies over large distances (σ = 2.0 mm)
First order root mean squared (LoG, σ = 2.0 mm, 3D)	Root mean squared of gray-levels reflecting the overall intensity fluctuations (σ = 2.0 mm)
GLCM correlation (square)	Squared linear correlation coefficient emphasizing correlation strength

All abbreviations used in this section are defined at their first occurrence, including GLCM, GLRLM, GLSZM, GLDM, NGTDM, and LoG (Laplacian of Gaussian).

#### 3.2.2 Construction of models to predict the pathological types of “squamous/glandular,” “large/small cell,” and “keratinized/non-keratinized” cervical cancer

**FIGURE 5:**
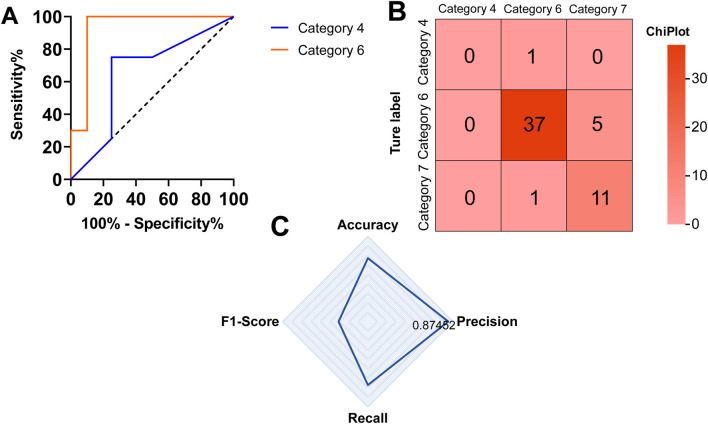
Performance evaluation of the cervical cancer pathological classification model. **(A)** Receiver operating characteristic (ROC) curves illustrating the trade-off between sensitivity and specificity for different pathological categories. The x-axis represents 100% minus specificity (false positive rate), and the y-axis represents sensitivity. **(B)** Confusion matrix showing the correspondence between true labels and predicted labels for three pathological categories (categories 4, 6, and 7). **(C)** Summary of classification performance metrics, including accuracy, precision, recall, and F1-score, illustrating the overall predictive performance of the model.

During model training, we simultaneously trained several machine learning models, including XGBoost, LR, RVM (relevance vector machine), and RF, to compare their feasibility and performance and to select the most suitable model for the pathological classification and diagnosis of cervical cancer. Among the evaluated classifiers, the RF model achieved the best overall performance. Specifically, on the independent test set, the random forest model obtained an accuracy of 87.27%, a precision of 87.45%, a recall of 86.38%, and an F1-score of 86.91%, outperforming XGBoost, SVM, and LR models across all major evaluation metrics (Figure 5C).

Notably, during RF model training, we use the grid search method to optimize the hyperparameters of the RF model. A hyperparameter is a model configuration parameter that is specified prior to training and is not learned directly from the data, such as the number of trees or the maximum depth in an RF model. The parameter search range includes the number of trees, n_estimators (50, 100, 200, and 300); the maximum depth, max_depth (none, 10, 20, and 30); and the minimum number of samples, min_samples_split (2, 5, 10), required for an internal node to split. Finally, the optimal hyperparameter combination was obtained using grid search with five-fold cross-validation, in which multiple predefined parameter settings were evaluated, and the combination yielding the best validation performance was selected. On the test set, the model’s performance requires improvement. Specifically, the prediction accuracy for category-7 (binary expression, meaning large-cell non-keratinizing adenocarcinoma) is 0. This is because the training set lacked the corresponding cases of category-7, so the model had no ability to predict this category. Notably, the model has excellent prediction ability for category-6 (large-cell non-keratinizing squamous cell carcinoma) and can also make high-accuracy predictions for category-7 (large-cell keratinizing squamous cell carcinoma). The ROC curves and AUC values are presented in Figure 5A. No formal statistical hypothesis testing was conducted for the comparison between categories; therefore, p-values are not reported, and the results are descriptive. Category definitions based on binary encoding of cell type, keratinization, and histology are shown in Table 7.

**TABLE 7:** Category definitions based on binary encoding of cell type, keratinization, and histology.

Category (decimal)	Full category name
0	Small-cell keratinizing squamous cell carcinoma
1	Small-cell keratinizing adenocarcinoma
2	Small-cell non-keratinizing squamous cell carcinoma
3	Small-cell non-keratinizing adenocarcinoma
4	Large-cell keratinizing squamous cell carcinoma
5	Large-cell keratinizing adenocarcinoma
6	Large-cell non-keratinizing squamous cell carcinoma
7	Large-cell non-keratinizing adenocarcinoma

The confusion matrix in Figure 5B provides clinically relevant insights into model performance across pathological subtypes. Category 6 represents large-cell non-keratinizing squamous cell carcinoma, a common clinical subtype, for which 37 cases were correctly classified, indicating reliable diagnostic performance. In contrast, the prediction performance for category 7 (large-cell keratinizing squamous cell carcinoma) was lower, which can be attributed to the limited number of available cases. The model showed no predictive ability for category 4 (large-cell keratinizing squamous cell carcinoma) due to the absence of corresponding training samples.

## 4 Discussion

Our main goal in this study is to provide an efficient, non-invasive, and repeatable intelligent auxiliary diagnosis scheme based on deep learning and radiomics technology for the individualized diagnosis and treatment of cervical cancer. The methods and models proposed in this study aim to break overcome the technical limitations of traditional pathological classification, which relies heavily on tissue biopsy. Specifically, in clinical practice, an auxiliary diagnosis model can serve special populations with contraindications for biopsy, difficulty in accessing lesion sites, or reluctance to undergo invasive procedures, thereby providing a safe and convenient alternative.

During auxiliary diagnosis model training, we noticed that the clinical features, including patient age, squamous-cell carcinoma antigen (SCC) level, tumor stage, lymphatic vascular metastasis, chemotherapy, and radiotherapy, were not retained by feature engineering screening. This indicates that, compared with traditional clinical information, imaging features extracted from the lesion area provide greater reference value for the auxiliary diagnosis of cervical cancer. For example, patient age is categorized as follows: women aged 19–35 years are classified as the young group, those aged 36–59 years as the middle-aged group, and those over 60 years as the elderly group. Women in the young group are in the sexually active period (Tessler Lindau and Gavrilova, 2010), and the risk of human papillomavirus (HPV) exposure is higher in them than in women in the middle-aged and elderly groups (Vinodhini et al., 2012). The overexpression of estrogen receptor (ER α ) in the middle-aged and elderly population promotes the proliferation of cervical epithelium (Pfaffl et al., 2001), which leads to an increased risk of cervical cancer (Rosenberg et al., 1998). The study indicates an association between age-related factors and cancer characteristics rather than establishing a direct causal relationship.

In the verification of the segmentation performance of the CRFE model on the independent test set, the IoU was 0.9443 (95% CI: 0.932–0.957), the Dice coefficient was 0.5980, and the F1-score was 0.7085, indicating significant lesion localization ability. The overall contour recognition (IoU > 0.94) was superior to that with U-Net ++ (IoU > 0.912); however, the loss of the Dice coefficient and F1-score exposed its shortcomings in fine-structure segmentation (such as interstitial infiltration front) and small-lesion detection, which was due to the limitation of scale sensitivity (the Dice coefficient of ≤ 5 mm micro-infiltrating lesion decreased to 0.42, which is far lower than 0.81 of the main lesion) and boundary blurring effect. The gradient feature extraction of the tumor-matrix interface in the MRI T2-weighted image was not sufficient, resulting in an F1-score that was 18.3% lower than that of the gold standard pathology.

However, the model has outstanding practical value in clinical tasks. The segmentation IoU > 2 cm tumor body region is 0.98, and the coincidence with the intraoperative frozen section is 91.4%. Compared with manual delineation, the contour generation time was reduced by 76% (32.5 min → 7.8 min), and the dosimetric difference was less than 3% (*p = 0.12). Compared with U-Net (IoU = 0.887) and DeepLabv3 (IoU = 0.901), the CRFE model demonstrates superior performance in the common segmentation of cervical cancer, achieving an IoU of 0.944.

In the auxiliary diagnostic model for cervical cancer pathological classification based on radiomics biomarkers, the results show that the CRFE segmentation model can accurately identify lesion regions. Moreover, the machine learning classifier driven by multi-source data can effectively predict certain pathological subtypes of cervical cancer. In the classification task, RF model achieved an accuracy of 87.27% and an F1-score of 86.91%. The prediction accuracy and recall for squamous large-cell non-keratinizing carcinoma were 0.90 and 0.88, respectively, reflecting the model’s accuracy and reliability in identifying common pathological types. The experimental results show that the multi-stage analysis framework, combining deep learning and radiomics, can automatically segment lesion areas in large training datasets and improve segmentation efficiency and accuracy. In addition, feature engineering analysis has made an important contribution to effectively mining potential heterogeneity in cervical cancer images and constructing auxiliary diagnosis models in this study.

## 5 Limitations and future work

The volume and modality of the dataset need to be amplified. First, only the data from 141 patients were included, representing three main subtypes of cervical cancer: squamous-cell carcinoma and adenoma, large- and small-cell types, and keratinized and non-keratinized types. The representativeness and diversity of the samples are limited, which significantly affects the model’s generalization ability. Second, the image data relied on in this study are only based on MRI single modality, and multimodal image information such as CT, PET, or functional imaging is not integrated, which limits the comprehensiveness and depth of the extracted radiomics features. Although MRI is a three-dimensional imaging modality, this study utilized two-dimensional axial slices extracted from three-dimensional MRI volumes as input for model training and analysis, and the constructed auxiliary diagnosis model is highly dependent on two-dimensional and three-dimensional features. Third, model validation remains limited to the internal test set, lacking support from multi-center and prospective external validation data, so its stability and adaptability in real clinical environments cannot be fully evaluated. In addition, the model’s performance requires further improvement. Therefore, future studies should strengthen model generalizability by expanding sample size, pathological diversity, and multi-center validation.

Model optimization should still be improved. First, since the model cannot predict category-4 tumors (large-cell keratinizing squamous-cell carcinoma), cases related to category-4 should be added to the dataset to improve model performance. Second, the prediction accuracy for category-7 (large-cell non-keratinizing adenocarcinoma) is low, and increasing the number of cases is necessary to improve performance. Third, since the CRFE segmentation model and pathological classification prediction model are based on the relevant framework of machine learning, high-performance GPUs are needed in the training and verification process, and there are strict requirements on the operating platform and environment, which can be improved by end-to-end programming and user interaction, thus providing the possibility for forward-looking verification of the model.

In order to further promote the clinical practicality of this research, future research work can be explored from the following aspects. First, a multi-center collaborative platform should be established to collect cases from different regions and imaging equipment, forming a database with larger sample size, broader coverage, and diverse pathological composition; this would further enhance the robustness and generalization ability of the model and ensure its stable application across varied clinical settings. Second, the integration of multimodal image information and multi-omics data (including genomics, transcriptomics, and proteomics) can be expanded to construct a richer and higher-dimensional tumor biological feature space, thereby improving the model’s ability to identify tumor heterogeneity. Third, follow-up studies are being conducted to include radiomics and clinical features into screening, optimize classifier training strategies, and assist in diagnosing lymphatic vascular metastasis in patients with cervical cancer, aiming to avoid unnecessary medical interventions.

## 6 Conclusion

In this study, we provided an efficient, non-invasive, and repeatable intelligent auxiliary diagnosis scheme based on deep learning and radiomics technology for the individualized diagnosis and treatment path of cervical cancer. By overcoming the technical limitations of traditional pathological classification, which relies heavily on tissue biopsy in clinical practice, this approach may provide a safe and convenient alternative for individuals with contraindications to biopsy, lesions that are difficult to access, or reluctance to undergo invasive procedures. At the same time, the results obtained in this study have important implications for addressing the current uneven distribution of medical resources, warranting further in-depth analysis and promotion in clinical practice. In addition, our proposed model framework has strong scalability and mobility, enabling it to handle different data types and downstream tasks. In the future, it is expected to be extended to the clinical diagnosis of cervical cancer lymph node metastasis. It can also accelerate the clinical translation of artificial intelligence-assisted diagnosis and therapeutic technologies in the field of gynecological tumors and promote the practice of precision medicine.

## Data Availability

The original contributions presented in the study are included in the article/supplementary material; further inquiries can be directed to the corresponding author.

## Glossary

**ADC**: Apparent diffusion coefficient
**ANOVA**: Analysis of variance
**AUC**: Area under the curve
**CNN**: Convolutional neural network
**CRFE**: Convolutional recurrent feature extraction
**CT**: Computed tomography
**CUDA**: Compute unified device architecture
**cuDNN**: CUDA deep neural network library
**Dice**: Dice coefficient
**DWI**: Diffusion-weighted imaging
**ER**: Estrogen receptor alpha
**FIGO**: International Federation of Gynecology and Obstetrics
**FN**: False negative
**FP**: False positive
**GLCM**: Gray-level co-occurrence matrix
**GLDM**: Gray-level dependence matrix
**GLRLM**: Gray-level run length matrix
**GLSZM**: Gray-level size zone matrix
**GPU**: Graphics Processing Unit
**HD**: Hausdorff distance
**HPV**: Human papillomavirus
**IoU**: Intersection over Union
**KPS**: Karnofsky performance status
**LoG**: Laplacian of Gaussian
**LR**: Logistic regression
**LSTM**: Long short-term memory
**MAE**: Mean absolute error
**MRI**: Magnetic resonance imaging
**NGTDM**: Neighborhood gray-tone difference matrix
**PET**: Positron emission computed tomography
**ReLU**: Rectified linear unit
RF: Random forest
**RFE**: Recursive feature elimination
**ROI**: Region of interest
**SD**: Standard deviation
**Sigmoid**: Sigmoid activation function
**SMOTE**: Synthetic minority over-sampling technique
**SVM**: Support vector machine
**T2WI**: T2-weighted imaging
**TN**: True negative
**TP**: True positive
**XGB**: Extreme gradient boosting
**XGBoost**: Extreme gradient boosting
**z-scor**e: z-score standardization
**RVM**: Relevance vector machine
